# FGF8 induces therapy resistance in neoadjuvantly radiated rectal cancer

**DOI:** 10.1007/s00432-018-2757-7

**Published:** 2018-10-01

**Authors:** Felix Harpain, Mohamed A. Ahmed, Xenia Hudec, Gerald Timelthaler, Gerd Jomrich, Leonhard Müllauer, Edgar Selzer, Wolfgang Dörr, Michael Bergmann, Klaus Holzmann, Bettina Grasl-Kraupp, Michael Grusch, Walter Berger, Brigitte Marian, Gerd R. Silberhumer

**Affiliations:** 1 Department of Medicine I, Institute of Cancer Research, Medical University of Vienna, Borschkegasse 8a, 1090 Vienna, Austria; 2 Department of Surgery, Medical University Vienna, Vienna, Austria; 3 Radiation Biology Department, National Center for Radiation Research and Technology, Egyptian Atomic Energy Authority, Nasr City, Cairo Egypt; 4 Department of Radiotherapy and Radiobiology, Medical University of Vienna, Vienna, Austria; 5 Christian Doppler Laboratory for Medical Radiation Research for Radiation Oncology, Medical University of Vienna, Vienna, Austria; 6 Clinical Institute of Pathology, Medical University Vienna, Vienna, Austria

**Keywords:** Rectal cancer, Neoadjuvant radiochemotherapy, Fibroblast growth factor 8, Survivin, Therapy response

## Abstract

**Purpose:**

Therapy response to neoadjuvant radiochemotherapy (nRCT) of locally advanced rectal cancer varies widely so that markers predicting response are urgently needed. Fibroblast growth factor (FGF) and FGF receptor (FGFR) signaling is involved in pro-survival signaling and thereby may result in radiation resistance.

**Methods:**

In a cohort of 43 rectal cancer patients, who received nRCT, we analyzed protein levels of FGF 8 and its downstream target Survivin by immunohistochemistry to assess their impact on nRCT response. In vitro resistance models were created by exposing colorectal cancer cell lines to fractionated irradiation and selecting long-term survivors.

**Results:**

Our findings revealed significantly higher FGF8 and Survivin staining scores in pre-treatment biopsies as well as in surgical specimens of non-responsive compared to responsive patients. Functional studies demonstrated dose-dependent induction of FGF8 mRNA expression in mismatch-incompetent DLD1 cells already after one dose of irradiation. Surviving clones after one or two series of radiation were more resistant to an additional radiation fraction than non-irradiated controls and showed a significant increase in expression of the FGF8 receptor FGFR3 and of Survivin on both the RNA and the protein levels.

**Conclusion:**

The results of this study suggest that FGF8 and Survivin contribute to radiation resistance in rectal cancer and may serve as markers to select patients who may not benefit from neoadjuvant radiotherapy.

**Electronic supplementary material:**

The online version of this article (10.1007/s00432-018-2757-7) contains supplementary material, which is available to authorized users.

## Introduction

For locally advanced rectal cancers (LARC), neoadjuvant radiochemotherapy (nRCT) followed by surgery according to the principles of total mesorectal excision is the standard-of-care approach that can achieve downstaging rates as high as 28–62% of the cases, improved local control, higher rates of resectability and sphincter-sparing procedures (Villemure et al. 2003). Local pathological response to radiochemotherapy impacts on outcome rates after nRCT, irrespective of tumor stage (Mohindra et al. 2002). However, complete response rates vary between only 5–25%, while around 50% of rectal cancer patients respond poorly or are non-responsive to nRCT (Park et al. 2012; Ferlay et al. 2015; Lorimer et al. 2017). Predictive markers are needed that allow an accurate selection of patients, who will most likely benefit from nRCT or those who could be spared therapy that is associated with high comorbidities.

Aberrant fibroblast growth factor (FGF) and FGF receptor (FGFR) signaling has been reported for colorectal cancer and supports tumor cell survival (Shimokawa et al. 2003; Sonvilla et al. 2008, 2010; Heinzle et al. 2012). Recently, FGFR4 has been described to inhibit nRCT response in rectal cancer by stimulation of homologous recombination repair of radiation-induced DNA damage (Ahmed et al. 2016). However, this mechanism only works for mismatch repair (MMR)-competent tumor cells, leaving the question whether additional survival mechanisms operate in MMR-deficient lesions.

FGF8, an essential factor during embryonic development (Tickle and Munsterberg 2001; Brewer et al. 2016), is overexpressed in several tumor types including prostate, ovarian, breast, hepatocellular and colorectal cancers (Tanaka et al. 1998; Mattila and Harkonen 2007; Gauglhofer et al. 2011; Liu et al. 2014). The factor stimulates anti-apoptotic pathways (Zhang et al. 2006; Mattila and Harkonen 2007) and prevents tumor cell death (Gauglhofer et al. 2011) mediated by the IIIc-splice variants of receptors FGFR1-3 and FGFR4 (Dammann et al. 2015b). Up-regulation of FGF8 and FGFR3 was observed in CRC cells and xenografts receiving irinotecan-based chemotherapy, which led to induction of chemotherapy resistance (Erdem et al. 2017). In hepatocellular carcinoma, FGF8 is involved in resistance to epidermal growth factor receptor targeting therapy (Pei et al. 2017).

To investigate whether FGF8-dependent survival signaling is involved in nRCT resistance in rectal cancer, expression levels of FGF8 and the anti-apoptotic protein Survivin were analyzed using immunohistochemistry (IHC) in human rectal cancer tissue obtained from pre-nRCT biopsies and surgical specimens. Radiation-resistant cell line models were used to assess the role of FGF8 anti-apoptotic response at a molecular level.

## Materials and methods

### Patients and clinical samples

Biopsy specimens were collected retrospectively from the pathology department from 43 patients with rectal cancer who received nRCT at the General Hospital of Vienna during the years 2012–2014. The patients gave their informed consent and biopsies were taken during colonoscopic examination before preoperative radiotherapy. Tumor specimens were collected at surgery. The study protocol was approved by the ethics committee of the Medical University of Vienna (EK-1350/2016). All patients received a neoadjuvant regimen of capecitabine plus a total of 50.4 Gy radiation dose. Response to radiotherapy was determined by histopathological examination of surgically resected specimens and classified according to the amount of viable tumor cells in the resected tissue (Dworak et al. 1997).

### Immunohistochemistry and scoring

Immunohistochemistry (IHC) was performed on paraffin-embedded sections from human rectal cancer tissue as described previously (Dammann et al. 2015a). Details are given in supplemental materials (Supplementary Table 2).

Scoring was performed by two blinded investigators (F. Harpain, G. Jomrich) as previously described (Dammann et al. 2015b). Points were given for staining intensity from 0 to 3 and for the percentage of positively stained epithelial cells from 0 to 4, 0 (< 1%), 1 (1–10%), 2 (10–50%), 3 (51–80%), and 4 (> 80%) in four separate fields of view. An immunoreactivity score (IRS) was generated by multiplication of the mean intensity and percentage scores. Scores greater or equal to 6 were considered as “high”, scores smaller than 6 considered as “low”.

### Cell lines

HCT116 and HT29 were obtained from the American Type Culture Collection. DLD1 was obtained from European Culture Collections. All cell lines were authenticated by Eurofins (Vienna, Austria). They were kept under standard culture conditions (5% CO_2_ at 37 °C) using minimal essential medium containing 10% fetal calf serum (FCS) (Sigma-Aldrich, St. Louis, USA).

### Ionizing radiation and in vitro radiosensitivity assay

Cells were irradiated using a Co-60 radiotherapy unit (Theratron 760, Theratronics, Ottawa, Canada). The surviving fraction of cells was determined by the clonogenic assay and calculated relative to the non-irradiated control (Franken et al. 2006).

For the selection of surviving cell clones, irradiation was performed using one or two series of 5 × 2 Gy doses of γ-radiation over a week each. After the final radiation dose, cultures were maintained for 10 days to permit the growth of long-term-surviving clones. Clones were harvested and pooled for analysis.

### RNA isolation and quantitative real-time PCR assay

Total RNA was isolated using Trifast (PeqLab, Germany) according to the manufacturer’s instructions, reversely transcribed and cDNA amplified using TaqMan-based assay performed on an ABI 7500 fast real-time PCR system (Applied Biosystems, Foster City, California, USA), as previously described (Sonvilla et al. 2010). The TaqMan kits used are listed in Supplementary Table S3.

### Protein isolation and western blotting

Proteins were extracted using HEPES lysis buffer supplemented with protease inhibitor cocktail (Complete—Roche, Germany) and phosphatase inhibitors and analyzed by western blotting using antibodies listed in Supplementary Table S2. Detection was performed using ECL Western Blot Detection Reagents (GE Healthcare).

### Statistical analysis

Statistical analysis was performed using SPSS (version 24.0). Immunoreactivity scores were analyzed using an independent sample two-tailed *t* test. Pearson’s *χ*^2^ test was used for analyzing the association between FGF8/Survivin expression and clinicopathologic parameters. *P* values < 0.05 were considered significant (*p < 0.05, **p < 0.01, ***p < 0.001). All data are expressed as mean ± standard deviation unless otherwise stated.

## Results

### Patient and tumor characteristics

43 patients receiving nRCT were included in the study. Patient characteristics including TNM staging at diagnosis and after surgery are given in Table 1. The patients were 26% female and 74% male. Median age was 68 years. The majority suffered from locally advanced tumors (40/43 patients, 93%) and positive lymph node involvement (34/43 patients, 79.1%).

**Table 1:** Patient characteristics

Age, median (IQR)	68 (26–90)
Sex, *n* (%)	
Female	11 (25.6)
Male	32 (74.4)
Pre-treatment staging, *n* (%)	
T1, 2	4 (9.3)
T3, 4	39 (90.7)
N0	9 (20.9)
N1, 2	34 (79.1)
Stages I and II	9 (7.0)
Stage III	34 (93.0)
Post-treatment staging, *n* (%)	
ypT1, 2	15 (34.9)
ypT3, 4	22 (53.5)
ypN0	29 (67.4)
ypN1, 2	14 (32.5)
Stage 0	5 (11.6)
Stages I and II	25 (58.1)
Stage III	13 (30.2)

nRCT resulted in reduction of tumor size in 19 patients (44.2%) and in a decrease of node involvement in 26 patients (60.5%). Twenty-three patients (53.5%) were downstaged. Complete remission (stage 0) was observed in five cases (11.6%). Pathological response to neoadjuvant therapy was determined based on the presence of viable tumor cells in the surgical specimens (Dworak et al. 1997). Details are described in Supplementary Materials (Supplementary Fig. 1). Complete pathological response (grade 4) was found in five cases (11.6%); 51.2% of specimens showed moderate to strong response (grades 2 and 3); 37.2% had no or insufficient response (grades 0 and 1). For the purpose of differential analysis, response grades 0–1 were classified as non-responders as opposed to the responders with grades 2–4.

### FGF8/Survivin expression correlated with poor local response to neoadjuvant radiochemotherapy in rectal cancer patients

Tissue sections obtained from pre-treatment biopsies were stained for FGF8 and Survivin using standard IHC methods. Representative examples of staining for both markers are shown in Supplementary Fig. 2. Staining was scored as described in “Materials and methods”. FGF8 and Survivin were assessed separately and each was grouped according to their IRS in high and low expressers (Table 2). No statistically significant association was observed between IRS and gender, age or pre-treatment and post-treatment staging for either marker.

**Table 2:** FGF8 and Survivin and their correlation to clinicopathological characteristics and response of rectal cancer patients treated with neoadjuvant radiochemotherapy

	FGF8 expression		*P* value	Survivin expression		*P* value
	Negative-weak^a^	Moderate-strong^a^		Negative-weak^a^	Moderate-strong^a^	
Median age, years	67.50	69.00	0.96	68.00	68.50	0.38^b^
Sex, *n* (%)						
Women	5 (28)	6 (24)	0.78	3 (20)	8 (29)	0.54^c^
Men	13 (72)	19 (76)		12 (80)	20 (71)	
Pre-treatment grading and staging, *n* (%)						
Depth of invasion, *n* (%)						
T1, 2	1 (6)	3 (12)	0.66	0 (0)	4 (14)	0.30^(c)^
T3, 4	17 (94)	22 (88)		15 (100)	24 (86)	
Lymph node metastasis, *n* (%)						
N0	3 (17)	6 (24)	0.84	2 (13)	7 (25)	0.41^c^
N1, 2	15 (83)	19 (76)		13 (87)	21 (75)	
TNM stage, *n* (%)						
Stages I and II	3 (17)	6 (24)	0.84	2 (13)	7 (25)	0.41^c^
Stage III	15 (83)	19 (76)		13 (87)	21 (75)	
Post-treatment grading and staging, *n* (%)						
Depth of invasion, *n* (%)						
ypTx, T1, 2	9 (50)	11 (44)	0.27	6 (40)	14 (50)	0.61^c^
ypT3, 4	9 (50)	14 (56)		9 (60)	14 (50)	
Lymph node metastasis, *n* (%)						
ypN0	14 (78)	15 (60)	0.42	9 (60)	20 (71)	0.31^c^
ypN1, 2	4 (22)	10 (40)		6 (40)	8 (29)	
TNM stage, *n* (%)						
Stage 0	4 (22)	1 (4)	0.17	2 (13)	3 (11)	0.71^c^
Stages I and II	11 (61)	14 (56)		7 (47)	18 (64)	
Stage III	3 (17)	10 (40)		6 (40)	7 (25)	
Therapy response, *n* (%)^d^						
Strong response (2–4)	16 (89)	11 (44)	0.003	13 (86)	14 (50)	0.02^c^
Weak or no response (0–1)	2 (11)	14 (56)		2 (13)	14 (50)	

^a^The classification was done according the immunoreactivity score (IRS) as described in “Materials and methods”: negative-weak (0–5), moderate-high (6–12)

^b^Correlation was determined by *t* test

^c^Correlation was determined by *χ*^2^ test

^d^Therapy response was determined according to the criteria of Dworak et al. (1997)

**Fig. 1:**
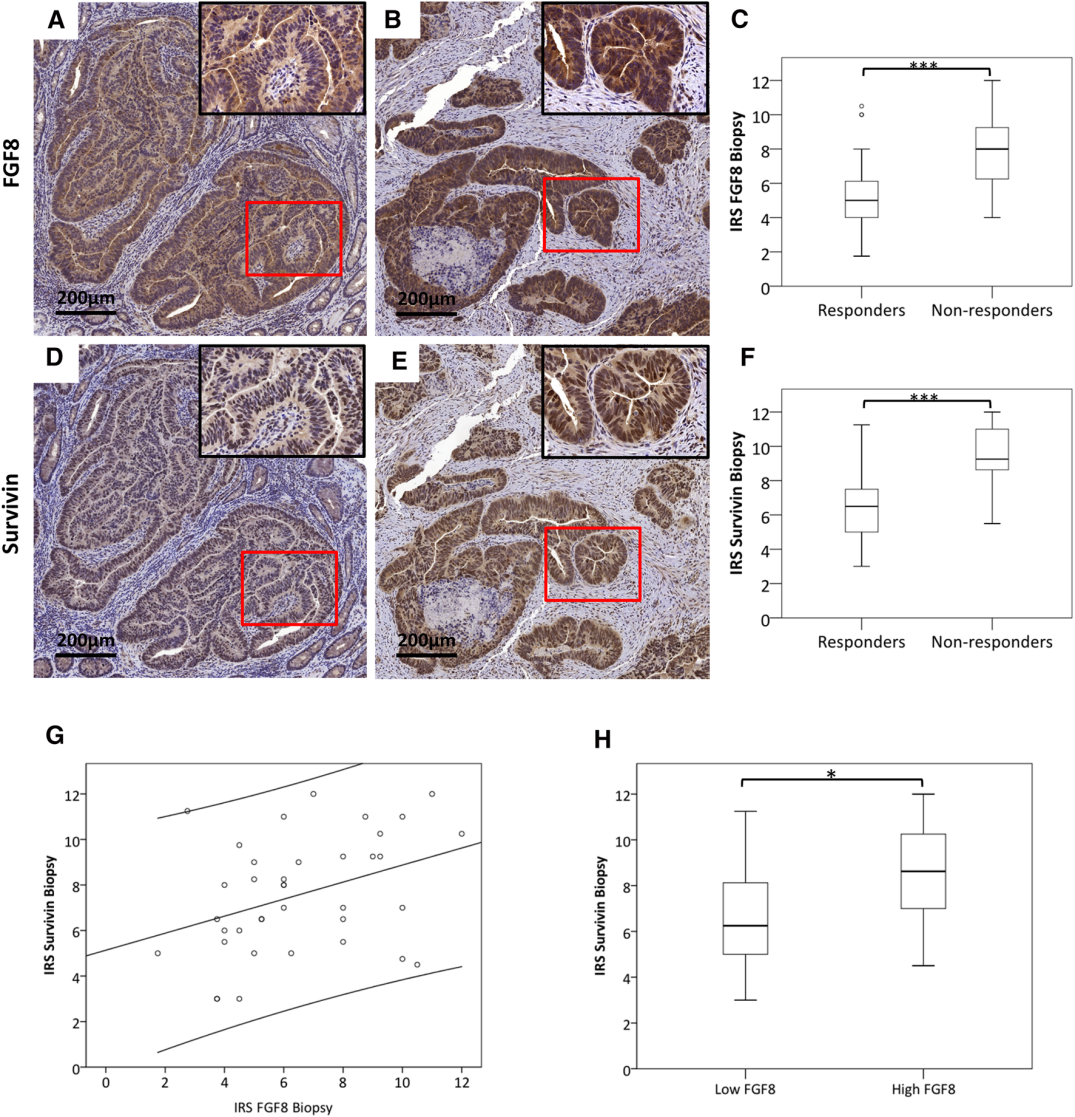
FGF8 and Survivin staining in responders and non-responders. Serial sections of pre-treatment biopsies were stained for FGF8 and Survivin. Patients were classified into responders and non-responders according to Dworak et al. (1997). **a, b, d, e** All specimens showed positive staining, but staining intensity differed between responders and non-responders. The bar corresponds to 200 µm. **c, f** Staining intensity was scored as described in “Materials and methods” and compared between responders and non-responders for FGF8 (**b**) and Survivin (**c**). **g** Pearson correlation coefficient was computed for IRS values of FGF8 and Survivin. (*p* = 0.02; r^2^ = 0.14; y = 5, 13 + 0, 37x). **h** Specimens were grouped into high FGF8 and low FGF8 expression as described in Table 2 and their Survivin IRSs were analyzed by Students’ *t* test. *Statistical significance at *p* < 0.05. ***Statistical significance at *p* < 0.001

The only parameter that revealed a significant association with FGF8 and Survivin levels was therapy response: downstaging was achieved in 10 patients with high FGF8 and 13 with low FGF8 levels (*p* = 0.04 by *χ*^2^ test). 4 of the 5 patients, who showed complete clinical response (post-treatment stage 0), were low-FGF8. For statistical analysis, patients were grouped into responders (grade 2–4) and non-responders (grade 0–1). Sixteen of the 18 specimens (89%) in the low-FGF8 group were responders, while in the high-FGF8 group the responders were only 11 of the 25 patients (44%) which was statistically significant at *p* = 0.003 by *χ*^2^ test (Table 2). Also FGF8 IRS was significantly lower in responders as compared to non-responsive patients (*p* < 0.001) (Fig. 1a–c).

For Survivin, the situation was similar but not as clear-cut: only two of the five complete responders were in the low-Survivin group. 13 of the 15 patients (87%) in the low-Survivin group were responders, while in the high-Survivin group only 50% of the patients (14 of 28) were responders (*χ*^2^ test: *p* = 0.02; Table 2). IRS for Survivin was significantly higher in responding than in non-responding patients (Fig. 1d–f, *p* < 0.001).

Overall, IRS of Survivin and FGF8 were significantly correlated (r^2^ = 0.14, *p* = 0.02; Fig. 1g) and the IRS for Survivin was significantly higher in high-FGF8-expressing tumors as compared with low-FGF8-expressing ones (*p* < 0.05; Fig. 1h).

**Fig. 2:**
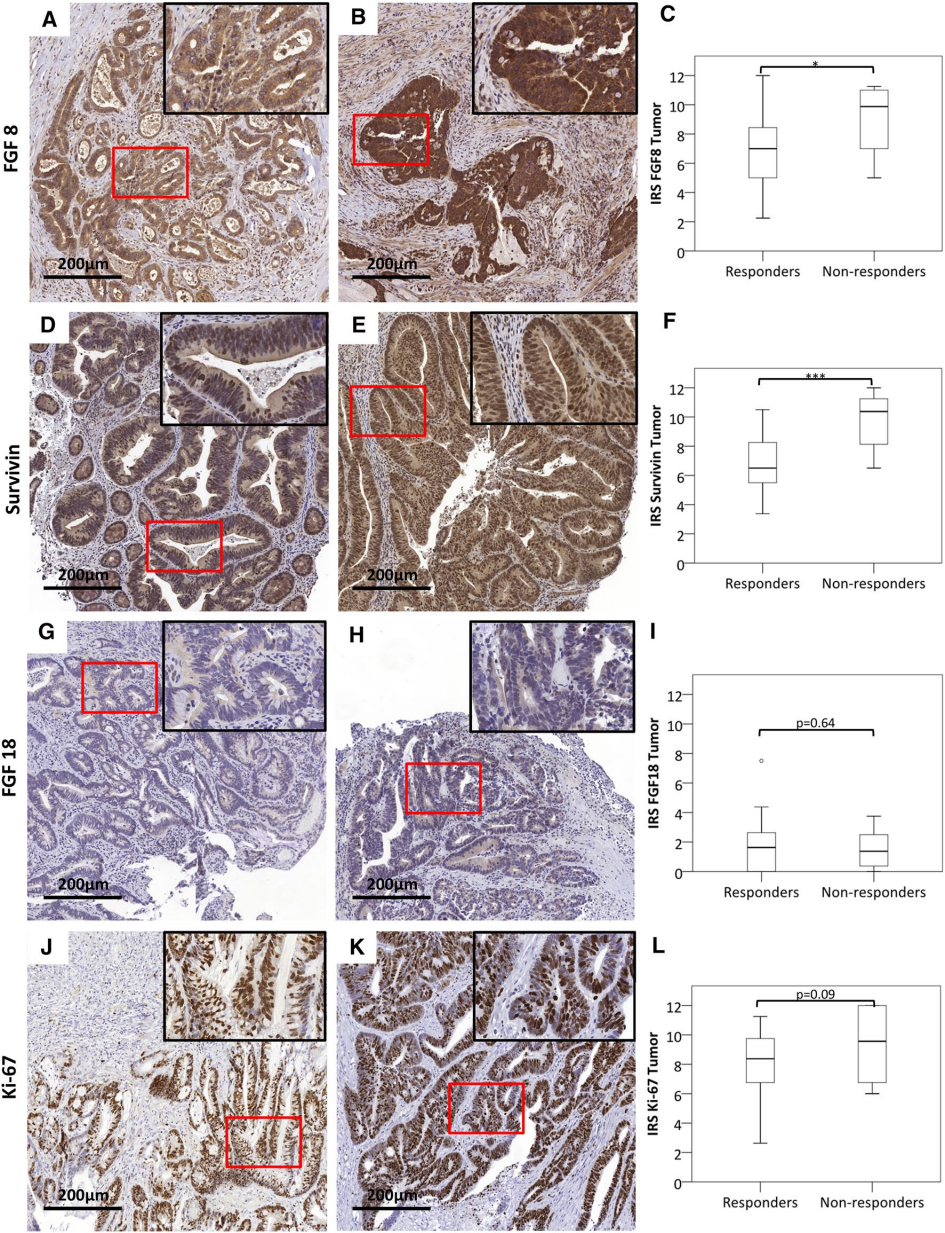
FGF8 and Survivin levels in surgical specimens of patients with incomplete response. Surgical specimens after nRCT of patients, who had a less than complete response were stained for FGF8 (**a**–**c**), Survivin (**d**–**f**), the alternative ligand FGF18 (**g**–**i**) and the proliferation marker Ki-67 (**j**–**l**). IRS was determined as described and compared between responders and non-responders (**c, f, i, l**). Size bar represents 200 µm. *Statistical significance at *p* < 0.05. ***Statistical significance at *p* < 0.001

To assess whether the FGF 8 and Survivin levels were retained in residual tumors after nRCT, surgical specimens were analyzed for those patients, who had a less than complete response. IRS was still higher in non-responders than in responders for both FGF8 (Fig. 2a–c) and Survivin (Fig. 2d–f). No difference was observed for the alternative ligand FGF18 (Fig. 2g–i) and the proliferation marker Ki-67 (Fig. 2j–l).

### Regulation of survivin and FGF8-dependent survival signaling in CRC cell lines

**Fig. 3:**
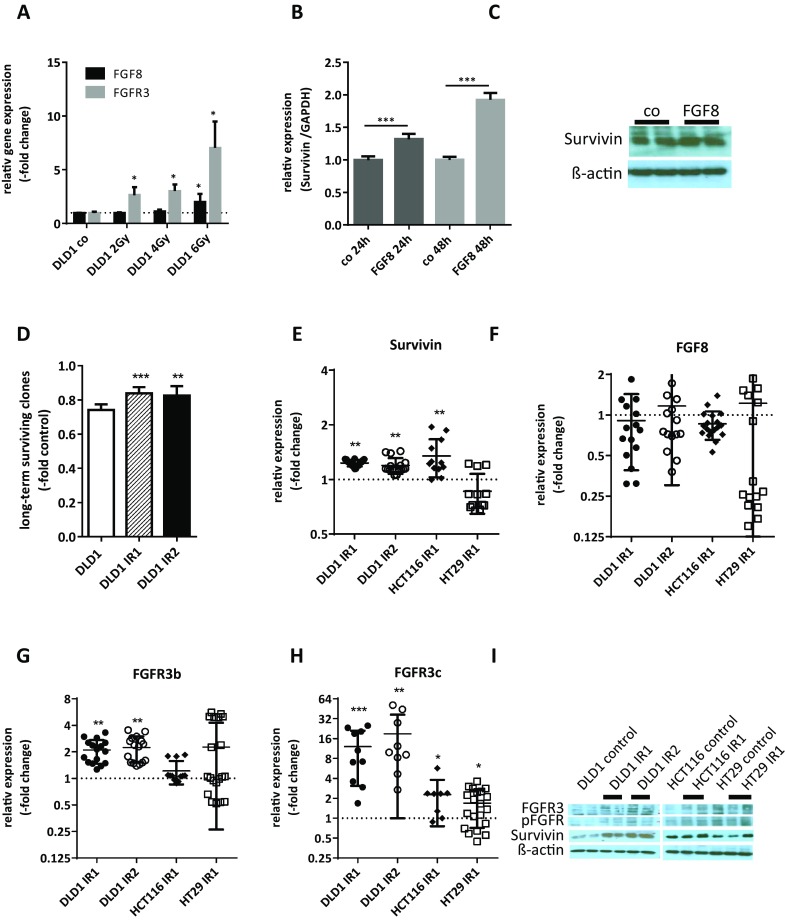
FGF8 and Survivin expressions in irradiated cell models. **a** DLD1 cells were irradiated with doses of γ-radiation of 2, 4 and 6 Gy. 24 h later, RNA was isolated and mRNA levels for FGF8 and FGFR3 were determined by qRT-PCR using TaqMan kits and the ΔΔct method. The house-keeping gene for normalization was GAPDH. Bars represent the mean ± SD of two independent experiments. **b, c** Logarithmically growing cultures of DLD1 cells were starved for 24 h before 10 ng/ml FGF8 was added to the medium. 24 and 48 h later, lysates were harvested for isolation of RNA and proteins. **b** Survivin mRNA levels were determined by qRT-PCR using TaqMan kits and the ΔΔct method. The house-keeping gene for normalization was GAPDH. **c** Survivin protein was determined by western blot. **d**–**i** Cultures of DLD1, HCT116, and HT29 cells were exposed to 5 (IR1) or 10 (IR2) doses of 2 Gy irradiation. Long-term-surviving cell clones were collected and clone pools were expanded for analysis. **d** DLD1 control cells, DLD1-IR1 and DLD1-IR2 were exposed to an additional dose of 2 Gy irradiation and radiation sensitivity was determined by the clonogenic assay. **e**–**h** mRNA levels in growing cultures were determined by qRT-PCR using TaqMan kits and the ΔΔct method with GAPDH as the house-keeping gene and non-irradiated cultures of equal density as the control. **e** Survivin, **f** FGF8, **g** FGFR3-IIIb, **h** FGFR3-IIIc. **i** Protein lysates were analyzed by western blotting using antibodies to Survivin, FGFR3 and p-FGFR. The figure shows representative panels from two independent experiments

To gain further insight into the role of FGF8-dependent survival signaling in CRC, we used HT29 cells (mismatch repair proficient), DLD1 and HCT116 cells (both mismatch repair deficient; Supplementary Table 1). With regard to FGF survival signaling, DLD1 and HT29 cells expressed low baseline levels of FGF8 and its receptor FGFR3 (Supplementary Fig. 3). When DLD1 cells were exposed to radiation, FGF8 and FGFR3 mRNA were induced in a dose-dependent manner within 24 h (Fig. 3a).

Addition of 10 ng/ml recombinant FGF8 to the culture medium of DLD1 caused a significant induction of Survivin mRNA expression after 24 and 48 h (Fig. 3b), while Survivin protein was only moderately elevated (Fig. 3c).

To obtain cell populations capable of surviving radiation treatment, all cell lines were subjected to one or two series of five radiation treatments (IR1 and IR2, respectively). Surviving clones were harvested 10 days after the final dose of radiation and clone pools were expanded for the isolation of mRNA and proteins. DLD1-IR cells selected in this manner were more resistant to an additional irradiation of 2 Gy than non-irradiated control cells (Fig. 3d). They displayed increased expression of Survivin on the mRNA (Fig. 3e; 1.23 ± 0.05-fold and 1.19 ± 0.12-fold in IR1 and IR2) as well as the protein level (Fig. 3i). FGF8 mRNA was not elevated (Fig. 3f), but enhanced FGFR3 expression was observed for both the IIIb-splice variant (Fig. 3g; 2.10 ± 0.16-fold and 2.25 ± 0.73-fold) and the IIIc-splice variant (Fig. 3h; 12.09 ± 9.00-fold and 18.94 ± 17.95-fold). FGFR3 protein and p-FGFR reactivities in the FGFR3 bands were higher in DLD1-IR1 and DLD1-IR2 cells as compared to controls (Fig. 3I).

Observations were similar for HCT116 cells: Survivin mRNA was 1.35 ± 0.32-fold higher in HCT116-IR1 than in HCT116 controls and Survivin protein was moderately elevated (Fig. 3e, i). With regard to FGFR3-IIIb, no further increase was detected, but FGFR3-IIIc was up-regulated 2.29 ± 1.53-fold in the HCT116-IR1 cells (Fig. 3g, h) and FGFR3 protein and p-FGFR were also higher (Fig. 3i). In HT29 cells, no induction of Survivin mRNA was seen and Survivin protein levels were variable in repeated experiments (Fig. 3e, i). In general, inter-experimental variation was high for HT29-IR1 cells, so that no statistically significant alterations could be found for FGFR3-IIIb and for FGFR3 protein and phosphorylation (Fig. 3g, i). Only FGFR3-IIIc was significantly increased even though the expression was decreased in some duplicate testings (Fig. 3h; 1.69 ± 0.97).

## Discussion

nRCT followed by surgical resection remains the standard therapy regimen for locally advanced rectal cancer patients (Villemure et al. 2003). However, therapy response varies widely (Ramzan et al. 2014) and radiotherapy may cause severe adverse events in irradiated patients. Thus, identifying biomarkers with the potential to predict therapy response is of utmost importance to enable the selection of a patient population, where benefits of radiotherapy clearly outbalance disadvantages.

Ionizing radiation mainly aims to damage and kill tumor cells by inducing DNA double-strand breaks (Sada et al. 2018). Cell cycle arrest and DNA damage repair through the homologous recombination or non-homologous end-joining pathway are physiological responses to the inflicted damage (Ramzan et al. 2014; Dammann et al. 2015a). If repair is unsuccessful, cells undergo programed cell death. Consequently, deregulation in repair and/or survival signaling impacts on radiation response and key regulators of this machinery may predict therapy response. Specifically, Survivin has previously been suggested as a sensitivity marker based on cell line models (Rodel et al. 2003) and FGFR-dependent signaling plays a central role due to its physiological functions (Turner and Grose 2010; Heinzle et al. 2011), especially in CRC (Sonvilla et al. 2008, 2010; Koneczny et al. 2015; Erdem et al. 2017).

In this study, protein levels of FGF8 and Survivin were strongly correlated with therapy response in a cohort of 43 rectal cancer patients indicating that they are promising candidates as predictive markers for nRCT response. Both markers strongly correlated with each other suggesting a mechanistic connection. This is conceivable based on our observation that FGF8 induced Survivin expression in DLD1 cells. The mechanism may be direct stimulation by FGFR-dependent survival signaling or indirect through the Wnt pathway, as Survivin is a Wnt-target gene (Widelitz 2005; Xu et al. 2017) and FGF signaling enhances β-catenin-dependent transcription activity (Koneczny et al. 2015).

In vitro, Survivin up-regulation was evident for DLD1 and HCT116 cells (MMR deficient), but not for HT29 cells (MMR competent), leaving the question whether this reaction may be specific for MMR-deficient tumors. However, a relatively large proportion of high-Survivin-expressing rectal tumors (28/43; 65%) was found in our cohort although in the literature MMR-deficient tumors in the rectum are described less frequently (de Rosa et al. 2016). Rather the difference may be due to the high degree of radiation resistance, inherent already in HT29 control cells, as they up-regulate FGFR4, which mediates efficient repair of radiation-induced DNA damage (Ahmed et al. 2016).

The impact of FGF survival signaling on radiation response was also demonstrated by analysis of irradiated cell line models. Specifically, DLD1 cells, whose baseline FGF signaling activity is low, showed strong induction of FGF8 and FGFR3 mRNAs immediately after exposure. For the induction of resistance mechanisms, a fractionated irradiation protocol was chosen to model therapeutic treatment schedules. Long-term survivors of this treatment were more resistant to a further radiation dose than non-irradiated control cells. In survivors of all three cell lines, FGFR3-IIIc, the receptor mediating survival signaling of FGF8 and FGF18 (Zhang et al. 2006; Sonvilla et al. 2010), was significantly increased on mRNA level as compared to non-irradiated control cells. In addition, FGFR3 protein was elevated and phosphorylated, indicating an active signaling state. Based on our results, up-regulation of FGF-dependent survival signaling has to be viewed as a general phenomenon in response to radiation damage in CRC cells, independent of MMR status. A similar effect was described in CRC cells exposed to irinotecan, which induced FGF8 and FGFR3 expression and thereby hampered therapy response (Erdem et al. 2017). Unfortunately, the relevant receptor (FGFR3-IIIc) cannot be used as a tissue marker, because its expression is generally too low (Sonvilla et al. 2010; Erdem et al. 2017). However, the receptor may provide a useful target for combination therapy. When applied after irinotecan treatment, an FGFR inhibitor had a synergistic effect (Erdem et al. 2017). FGFR inhibitors usually block FGFR1–3 and somehow less also FGFR4 (Heinzle et al. 2011, 2014). FGFR inhibition might be a useful strategy in the neoadjuvant setting for those patients that express high levels of FGF8.

In summary, the strong connection between FGF8/Survivin expression and therapy response in rectal tumor tissue underlines that they are predictive markers not restricted to mismatch repair-deficient tumors alone. Whereas the results of our previous study identified low levels of FGFR4 protein as a marker for good response to radiation (Ahmed et al. 2016), this study now demonstrates that high FGF8 and Survivin protein levels identify tumors that respond insufficiently or not at all.

## Electronic supplementary material

Below is the link to the electronic supplementary material.

Supplementary material 1 (PDF 314 KB)

## Abbreviations

CRC: Colorectal cancer
FGF: Fibroblast growth factor
FGFR: Fibroblast growth factor receptor
IHC: Immunohistochemistry
LARC: Locally advanced rectal cancer
nRCT: Neoadjuvant radiochemotherapy
