# Supplementary material for: FGF8 induces therapy resistance in neoadjuvantly radiated rectal cancer
Source: J Cancer Res Clin Oncol. 2018 Oct 1;145(1):77–86. doi: 10.1007/s00432-018-2757-7 (PMC6326005; doi:10.1007/s00432-018-2757-7)
Supplement: Supplementary file 1 — Supplementary material 1 (PDF 314 KB) [file 432_2018_2757_MOESM1_ESM.pdf]

FGF8 induces therapy resistance in neoadjuvantly radiated rectal cancer.

Felix Harpain, Mohamed A. Ahmed, Xenia Hudec, Gerald Timelthaler, Gerd Jomrich, Leonhard Müllauer, Edgar Selzer, Wolfgang Dörr, Michael Bergmann, Klaus Holzmann, Bettina Grasl-Kraupp, Michael Grusch, Walter Berger, Brigitte Marian, Gerd R. Silberhumer

Supplementary Material

1. **Patient characteristics and tumor regression grading**

Our study cohort consisted of 43 patients with rectal cancer who received nRCT at the General Hospital of Vienna during the years 2012-2014.

Pathological response was determined based on the presence of viable tumor cells in the tissue specimen after surgery according to the criteria established by Dworak et al. [1]. Specifically, 0 – no regression; 1 – dominant tumor mass with few signs of fibrosis; 2 – dominantly fibrotic material with few tumor cells or groups; 3 – very few tumor cells in fibrotic tissue; 4 – complete response - no tumor cells, only fibrotic mass. Response data are summarized in Supplementary Figure 1: 5 patients (11,63%) showed a complete response (grade 4), response grades 2 and 3 were observed in 7 and 15 patients. 6 patients had only a grade 1 response and 10 did not respond at all (grade 0).

Supplementary Figure 1: Therapy response.

Pathological response was determined based on the presence of viable tumor cells in the tissue specimen after surgery according to the criteria established by Dworak et al. [1].

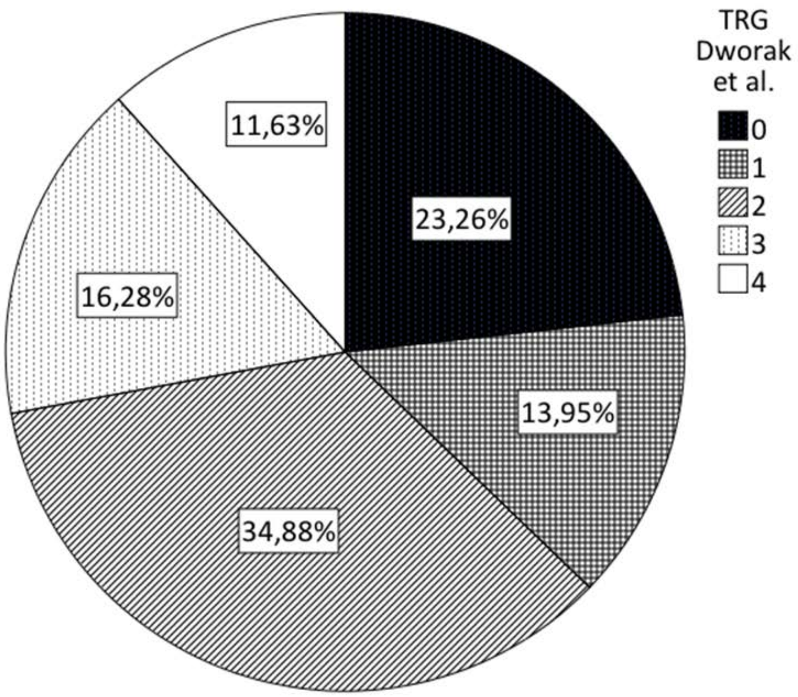

2. **Immunohistochemical analysis of patient material**

Both pre-treatment biopsies and post-treatment surgical specimens were collected from 43 patients diagnosed with rectal cancer who underwent nRCT at the general Hospital of Vienna in the years 2012-2014. FGF8 and Survivin were analyzed by immunohistochemistry and evaluated using an immunoreactivity score (IRS) that included points for staining intensity and for the percentage of positively stained cells (Supplementary Figure 2). Supplementary Figure 2 depicts typical stainings for FGF8 (Supplementary Figure 2A) and Survivin (Supplementary Figure 2B).

Supplementary Figure 2: Typical staining patterns for FGF8 and Survivin.

Serial sections were obtained from formalin-fixed paraffin-embedded surgical specimens of rectal tumors. FGF8 and Survivin were detected using the antibody dilutions listed in Supplementary Table 2 and peroxidase-coupled secondary antibodies. The figure shows typical examples of negative, weak, moderate and strong stainings for FGF8 (A) and Survivin (B). The bar represents 200µm.

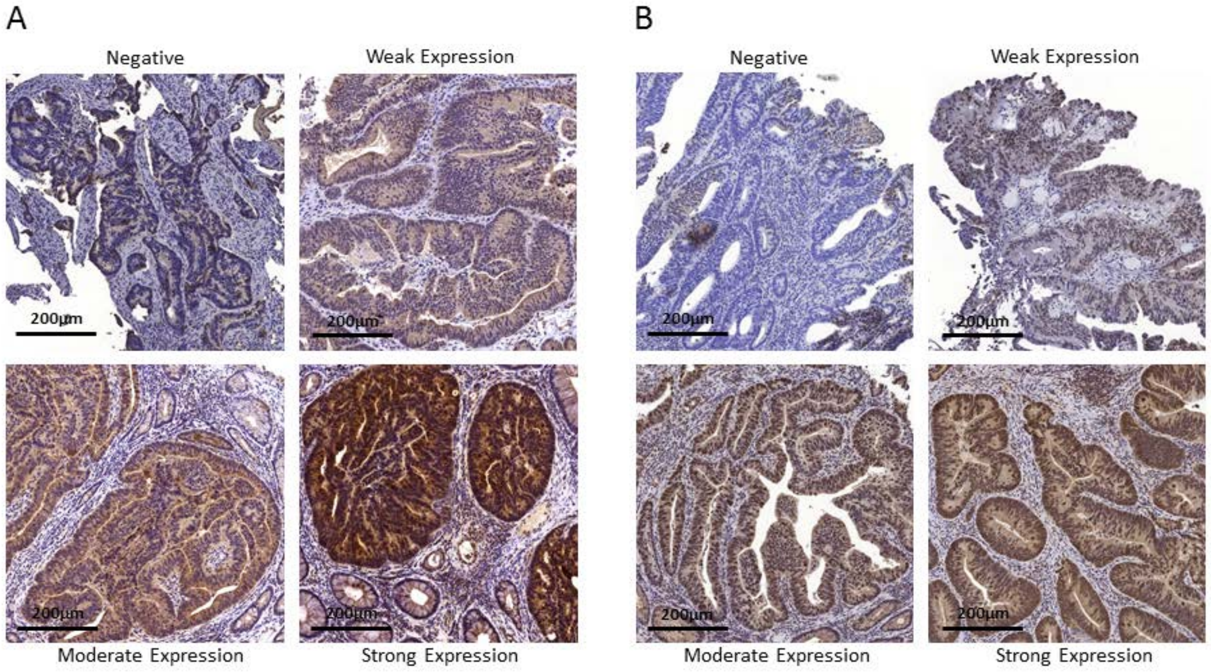

3. Cell line models

We used the mismatch repair-deficient cell lines HCT116 and DLD1, which are both sensitive to radiation-induced cell death. HT29 cells were included, because they are the radiation-resistant cell line model we used in our previous study [2]. The three cell lines differ in several genetic markers, most importantly p53 (Supplementary Table 1). In addition to their microsatellite instability (MSI), HCT116 and DLD1 have a wt-p53, in their MAP kinase pathway the mutation is in K-ras; in the TGFß-pathway the mutation is located in the TGFBR2 gene. By contrast, the microsatellite stable (MSS) cell line HT29 carries mutations in p53, B-raf and SMAD4.

Supplementary Table 1: Mutation status of cell line models [3].

	**HCT116**	**DLD1**	**HT29**
**MSS / MSI**	MSI	MSI	MSS
**Mismatch repair genes mutated**	MSH3	MLH1, MSH3, MSH6	none
**APC**	wt	mut	mut
**P53**	wt	wt	mut
**Ki-ras**	mut	mut	wt
**B-raf**	wt	wt	mut
**SMAD4**	wt	wt	mut
**TGFBR2**	mut	mut	wt

With regard to FGFR3-mediated survival signalling, we analyzed mRNA levels for FGF8, FGFR3-IIIb and FGFR3-IIIc from semi-confluent non-irradiated control cultures. The results show that DLD1 cells express the lowest levels of these genes, while HCT116 have the highest mRNA levels and HT29 cultures have moderate mRNA levels (Supplementary Figure 3).

Supplementary Figure 3: Baseline expression of FGF survival signaling-related genes.

RNA was isolated from semi-confluent, non-irradiated control cultures of DLD1, HCT116 and HT29 cells. mRNA levels of FGF8 (A) , FGFR3-IIIb (B) and FGFR3-IIIc (C) were determined using the Taqman kits listed in Supplementary Table 3.

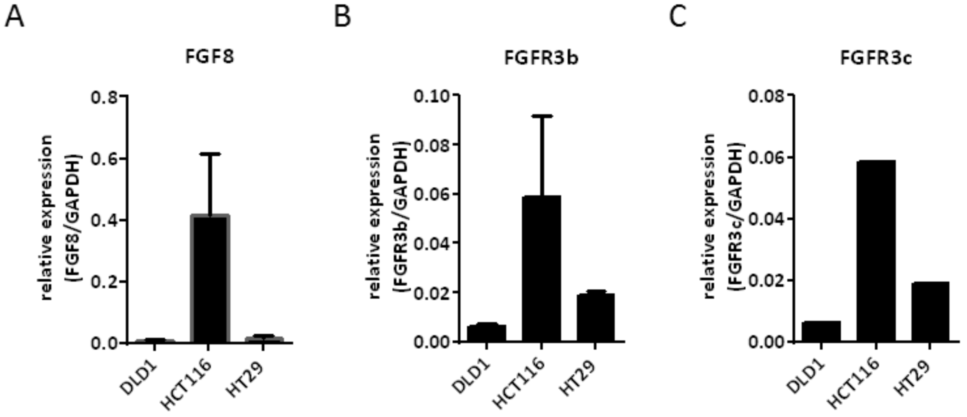

4. Conditions used for protein and RNA analysis

Antibodies used for immunohistochemistry (IHC) or western blot are listed in Supplementary Table 2.

Supplementary Table 2: Antibodies used in the study.

Protein	Company	Product number	Dilution	Method
Survivin	Novus Biologicals	NB500-201	1:500, 1:1000	IHC, WB
FGF8	Abcam	Ab203030	1:400	IHC
FGF18	Abcam	Ab139387	1:500	IHC
FGFR3 total	R&D	Mab7662	1:2000	WB
pFGFR	Cell Signaling Technology	3471	1:500	WB
ß-actin	Sigma	A-4700	1:5000	WB
Ki-67	Dako	M7240	1:100	IHC

IHC = immunohistochemistry, WB = western blot

mRNA levels were analyzed by quantitative RT-PCR using Taqman Kits (Supplementary Table 3) and the $\Delta\Delta c_T$-method.

Supplementary Table 3: Taqman Kits used for analysis.

Gene	Kit #
Survivin	Hs00977611_g1
FGF8	Hs00171832_m1
FGFR3-IIIb	Hs01005396_m1
FGFR3-IIIc	Hs00997397_m1
GAPDH	Hs99999905_m1

REFERENCES

1. Dworak, O., L. Keilholz, and A. Hoffmann, *Pathological features of rectal cancer after preoperative radiochemotherapy.* Int J Colorectal Dis, 1997. **12**(1): p. 19-23.
2. Ahmed, M.A., et al., *Fibroblast growth factor receptor 4 induced resistance to radiation therapy in colorectal cancer.* Oncotarget, 2016. **7**(43): p. 69976-69990.
3. Mouradov, D., et al., *Colorectal Cancer Cell Lines Are Representative Models of the Main Molecular Subtypes of Primary Cancer.* Cancer Research, 2014. **74**(12): p. 3238-3247.
